# A proposal for a novel impact factor as an alternative to the JCR impact factor

**DOI:** 10.1038/srep03410

**Published:** 2013-12-03

**Authors:** Zu-Guo Yang, Chun-Ting Zhang

**Affiliations:** 1 Library, Tianjin University, Tianjin 300072, China; 2 Department of Physics, Tianjin University, Tianjin 300072, China

## Abstract

One disadvantage of the JCR impact factor, the most commonly used assessment tool for ranking and evaluating scientific journals, is its inability in distinguishing among different shapes of citation distribution curves, leading to unfair evaluation of journals in some cases. This paper aims to put forward an alternative impact factor (IF′) that can properly reflect citation distributions. The two impact factors are linearly and positively correlated, and have roughly the same order of magnitude. Because of the ability of IF′ in distinguishing among different shapes of citation distribution curves, IF′ may properly reflect the academic performance of a scientific journal in a way that is different from the JCR impact factor with some unique features that reward journals with highly cited papers. Therefore, it is suggested that IF′ could be used to complement the JCR impact factor.

The concept of the impact factor was proposed by Garfield^1^ about 60 years ago. Since then, the impact factor has become the most commonly used assessment tool for ranking and evaluating scientific journals. However, the impact factor suffers from a number of shortcomings and limitations^2^,^3^,^4^,^5^. These shortcomings include: (i) Because the JCR impact factor is only an average citation received, it does not possess the ability to discriminate the shapes of citation distribution curves, leading to cases in which the same impact factor may correspond to different journals with different citation distributions. (ii) For the same reason, the JCR impact factor as an index of citations per publication usually rewards low productivity, and penalizes high productivity^6^. (iii) The JCR impact factor is relatively easily manipulated by increasing self-citations. On the other hand, Hirsch launched a new study direction of scientometrics by proposing a novel index, now known as the *h*-index^6^. The *h*-index was quickly adopted in various research areas. For example, the *h*-index or *h*-type indices were used for evaluating physicists^7^, evaluating the 100 most prolific economists^8^, and for evaluating chemical research groups correlated with peer judgment^9^. Recent studies of the *h*-index were reviewed^10^,^11^,^12^,^13^.

Meanwhile, a number of researchers explored the possibility of using the *h*-index to complement or correct the traditional impact factor. Indeed, the *h*-index or *h*-type indices were used to complement journal impact factors or to rank scientific journals. For example, Braun, Glänzel, & Schubert suggested that the *h*-type index may be a useful complement to journal impact factors^14^. Schubert and Glänzel performed a systematic analysis of *h*-type indices for journals^15^. A theoretical model of the dependence of *h*-type indices on the number of publications and the average citation rate was tested successfully on empirical samples of journal *h*-indices^15^. Vanclay ranked forestry journals using the *h*-index^16^. Relationship between the *h*-index and impact factor in the power-law model was studied by Egghe, Liang, & Rousseau^17^.

Despite these beneficial exploring researches, the *h*-index suffers from a number of inherent shortcomings. Probably, the most notable shortcoming of the *h*-index is that only *h-*squared citations can be inferred from the *h-*index, which completely ignores excess citations^18^ and *h-*tail citations^19^,^20^. The *h-*index by itself does not carry information for excess and *h-*tail citations, which can play an even more dominant role than the *h-*index in determining the shape of citation distribution curve. Ignoring the contributions from the excess and *h*-tail citations usually either under-estimates or over-estimates the academic performance of the scientist or the journal under study. To solve this problem, recently a new *h*-type index was proposed, called the *h*′-index^21^. One of the main merits of the *h*′-index is that it takes *h*-squared, excess and tail citations all into account. The *h*′-index possesses the ability to distinguish between different shapes of the citation distribution curves. The proposal of the *h*′-index inspired us to explore the possibility of using the *h*′-index to complement and correct the traditional impact factor, and this is the aim of the present paper.

## Results

### Derivation of an alternative impact factor, IF′

To complement the *h*-index^6^, the *e*-index^18^ and *t*-index^20^ were proposed. The *e*-index is the square root of the excess citations over *h*^2^ in the *h*-core. The *t*-index is the square root of the *h*-tail citations^19^. Therefore, the area under the citation distribution curve is divided by the *h-*index into three parts, representing the *h*^2^, the excess citations (*e*^2^) and *h-*tail citations (*t*^2^), respectively. To capture the main shape of the citation distribution curve, the head-tail ratio, denoted by *r*, was defined^21^

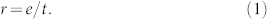
The three cases of *r* > 1, *r* = 1 and *r* < 1 correspond to three types of the citation distribution functions. The shapes of citation distribution functions for *r* > 1 are peaked, and for *r* < 1 the shapes of the citation functions are flat with a long tail, whereas for *r* = 1 the citation functions are roughly symmetrical with respect to the diagonal line of the coordinate system. A number of authors attempted to apply the *h*-index to complement or correct the JCR impact factor^14^,^15^,^16^. It should be pointed out that we cannot simply use the *h*-index to complement or correct the JCR impact factor. This is due to the fact that the *h-*index by itself does not carry information for the excess and *h-*tail citations, which can play an even more dominant role than the *h-*index in determining the shape of citation distribution curve. As pointed out previously^21^, when *r* > 1, especially *r* ≫ 1, the *h-*index under-estimates the academic performance of a scientific journal,whereas when *r* < 1, especially *r* ≪ 1, the *h-*index over-estimates the academic performance of a scientific journal. When *r* = 1, the *h-*index properly reflects the academic performance of a scientific journal.

To provide an alternative evaluation of the academic performance of a scientific journal, we propose a novel impact factor, denoted by *IF*′, to complement the JCR impact factor, defined by 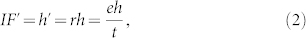
where *h*′ is the h′-index^21^.

The citations received by all papers in the *h-*core, denoted by *C_h-core_*, are 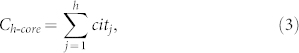
where *cit_j_* are the citations received by the *j*^th^ paper. Letting *e*^2^ denote the excess citations within the *h-*core, we find^18^

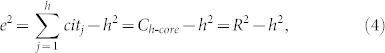
where *R* is the *R*-index^22^. So, 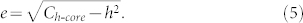
Meanwhile, the *t-*index was defined by^20^

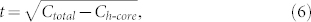
where *C_total_* is the number of total citations received by all papers published. Finally, we have 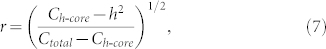
and 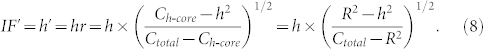
Based on the WoS database, all the published items for the 227 journals of biochemistry and molecular biology in 2009 and 2010 were retrieved. The retrieval resulted in 111,002 records, which were downloaded and saved as Excel files. Finally, we calculated the *h*-index, *e*-index and *t*-index of the 227 journals according to the citation data derived from the 111,002 records. Of note, all the calculations of the *h*-index, *e*-index and *t*-index were within a time window from the publication year of items to 2011. The parameter *C_total_* is the total citations received by all items published in 2009 and 2010 during the time window from the publication year of items to 2011. The parameter *C_h-core_* is the citations received by all items in the *h-*core of the citation distribution curve. Then IF′ can be calculated using eqs. (2) or (8). The JCR impact factor for the year 2011 is the ratio of the total citations received in 2011 by all items published in 2009 and 2010 over the total numbers of citable items published in 2009 and 2010^23^.

### Correlation between IF′ and the JCR impact factor

**Figure 1:**
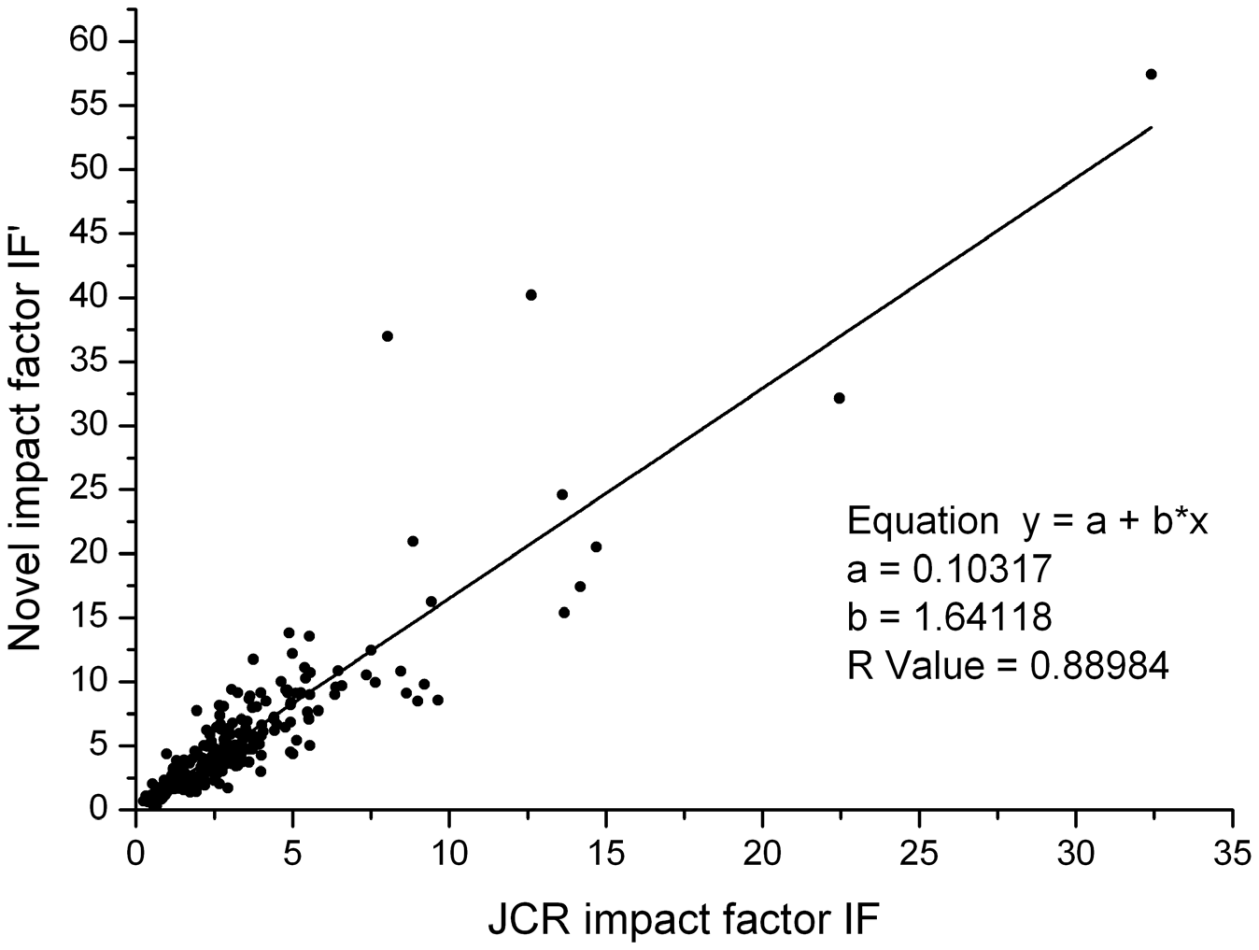
The correlation between IF′ and the JCR impact factor (IF). The related data of IFs and IF were derived from the JCR and the WoS for the 227 journals in the category of biochemistry and molecular biology. Note that IF′ and IF is highly linear and positively correlated, as reflected by the fact that the correlation coefficient is as high as 0.89. Of note, IF′ and IF have roughly the same order of magnitude.

Based on the data derived from the JCR and WoS, the related parameters including the total citations *C_total_*, the *h*-index, *e*-index, *t*-index, the head-tail ration *r* and IF′ are calculated for each of the 227 journals in the category of biochemistry and molecular biology. To save printing space, only the front ten journals in the alphabetic order are listed in Table 1. The related data for all the 227 journals are listed in the Appendix-1. As we can see from Table 1 that the head-tail ratio *r* for nine of the ten journals listed is all less than 1. Indeed, for almost all of the 227 journals listed in the Appendix-1, the values of *r* are less than 1. To study the relation between the two impact factors, the correlation between IF′ and the JCR impact factor is shown in Fig. 1. It is seen that IF′ is highly linearly and positively correlated with the JCR impact factor, as reflected by the fact that the correlation coefficient is as high as 0.89. Of note, IF′ and the JCR impact factor have roughly the same order of magnitude. The above two features make IF′ relatively easy to be accepted by the academic community.

### Applications of IF′

As an application, we show here the rankings of the 227 journals in the category of biochemistry and molecular biology based on IF′. For comparison, rankings are also provided based on traditional JCR impact factor. To save printing space, only the top ten journals are listed in Table 3. The rankings for all the 227 journals are listed in the Appendix-2. As we can see from Table 3 that the two rankings overlapped in 8 of the top 10 journals (80%), except that orders of some journals were different. This fact indicates that IF′ is basically consistent with the JCR impact factor, confirming the observation in Fig. 1. The famous journal Cell is clearly ranked No. 1 in both rankings. Of note, the journal Nucleic Acids Research is ranked No. 15 in the rankings based on the JCR impact factor, whereas it is ranked No. 3 in that based on IF′. On the contrary, the EMBO Journal is ranked No.10 in the JCR ranking, whereas it is ranked No. 24 in that based on IF′. Furthermore, the journal Current Biology is ranked No. 8 in the JCR-rankings, whereas it is ranked No. 40 in the rankings based on IF′. The variations in both ranks are determined mainly by their shapes of citation distribution curves. Of note, according to the data listed in the Appendeix-1, the head-tail ratios for the journals Nucleic Acids Research, EMBO Journal and Current Biology are 0.597, 0.245 and 0.231, respectively. It is obvious that the shape of the citation distribution curve for the journal Nucleic Acids Research is relatively sharp, whereas those for the journals EMBO Journal and Current Biology are relatively flat with a longer tail.

In the analysis above, we show that with these journals, IF′ has a one-to-one correspondence, whereas the JCR impact factor has a one-to-multiple correspondence. This is due to the fact that the JCR impact factor does not possess the ability to discriminate the shapes of the citation distribution functions, whereas the shapes of the citation distribution curves are properly taken into account by IF′. As a consequence, the JCR impact factor as an index of citations per publication usually rewards low productivity, and penalizes high productivity^6^.

## Discussion

**Figure 2:**
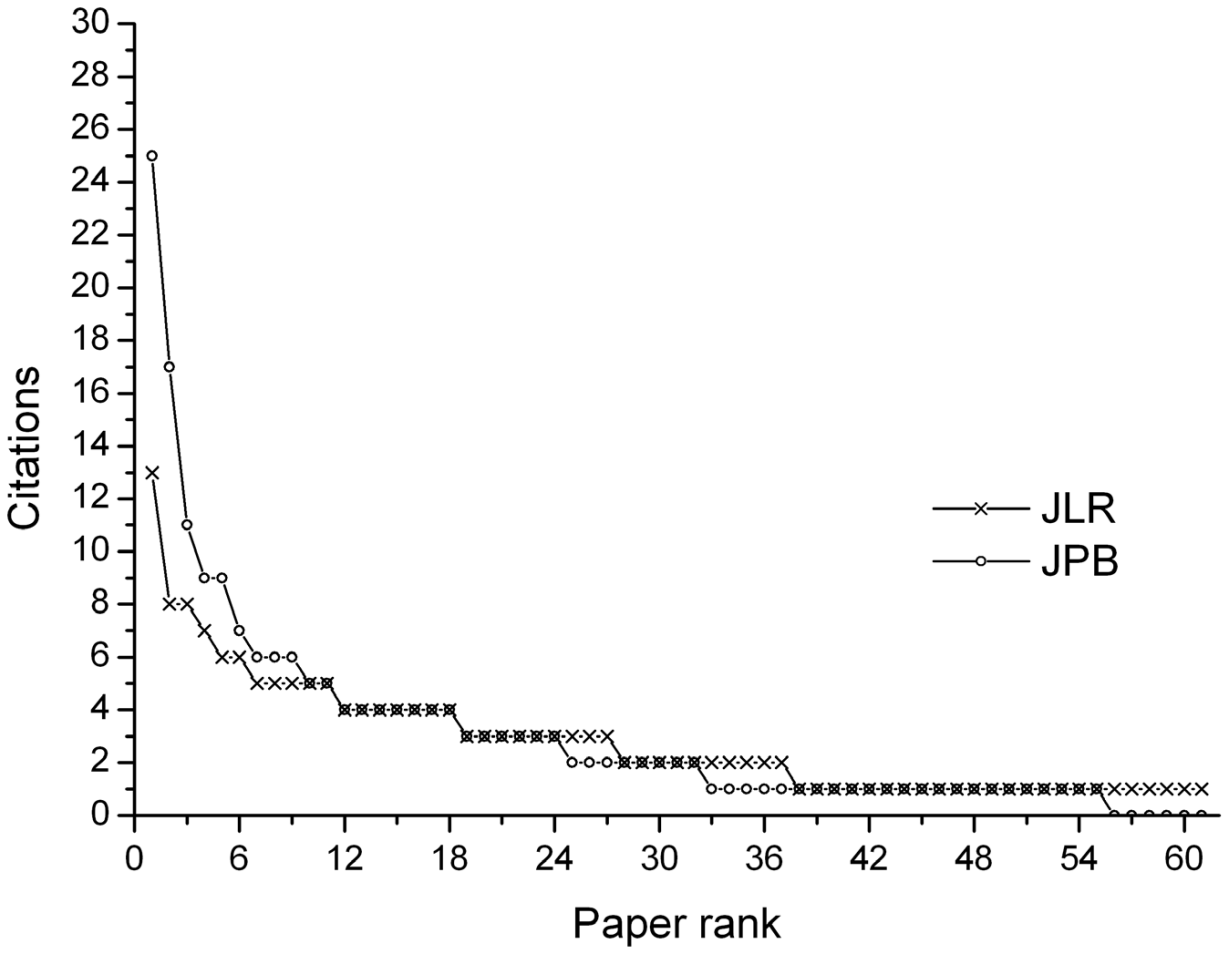
Comparison between two citation distribution curves for two journals with approximately equal JCR impact factor and number of publications. Note that the citation distribution curve for the journal JPB is relatively peaked, indicating that a part of its publications are highly cited, whereas that for the journal JLR is relatively flat, indicating that most of its publications are relatively lowly cited. Although the total citations are approximately equal, the academic performance of the two journals is different. The JCR impact factor cannot distinguish between the two journals, with almost identical JCR impact factor IF ≈ 1.7. On the contrary, IF′ possesses the ability to distinguish between the two journals with IF′ ≈ 3.6 and IF′ ≈ 1.8 for JPB and JLR, respectively.

The JCR impact factor is the averaged number of citations received per publication. As pointed out by Hirsch^6^, measuring the average citations per publication, as does the JCR impact factor, usually rewards low productivity, and penalizes high productivity. This is due to the fact that such indices do not possess the ability to distinguish between different shapes of citation distribution curves. To illustrate this point, let us consider two journals among the 227 journals. The first one is the Journal of Physiology and Biochemistry (JPB) and the second one is the Journal of Liposome Research (JLR). The JCR impact factor and the number of publications of both journals are roughly equal. The related data of the two journals are listed in Table 2. Their citation distribution curves are shown in Fig. 2. Note that the shapes of the two curves are different, as clearly shown in Fig. 2. The shape of the citation distribution curve for JPB is relatively sharp, whereas that for JLR is relatively flat. Unfortunately, the JCR impact factor cannot distinguish between the two cases, resulting in almost identical impact factor values (1.711 vs. 1.707). On the contrary, IF′ is able to distinguish between the academic performance of the two journals,resulting in the values of IF′ (3.658 vs.1.867).This example shows that IF′ results in distinct values for different journal citation distributions, whereas different citation distribution curves may correspond to the same JCR impact factor value. In other words, IF′ and journals have a relation of one-to-one correspondence, whereas the JCR impact factor and journals have a relation of one-to-multiple correspondence. Table 2 shows that in addition to the numbers of publication, the total citations, the average citations per publication (i.e., the JCR impact factor) and the *h*-indices are all roughly equal to each other for the two journals. Of note, the four indices mentioned above are all the single-number evaluation indices currently available and widely used nowadays. Unfortunately, none of them is capable of discriminating the academic performance of the two journals. On the contrary, IF′ (or the *h*′-index) is a single-number evaluation index that is able to distinguish between the academic performance of journals with different citation distribution. In addition to the two journals mentioned above, other three similar pairs of journals are also listed in Table 2.

Of note, the example above is a typical case, rather than a rare event, because we can find a number of similar cases, as shown in Table 2. Let us further consider two or more journals with roughly equal JCR impact factors, but the numbers of publication may be different. Such cases occur more frequently than the example shown above. Although their JCR impact factors are roughly equal, their citation distribution curves may be different, even quite different. The traditional JCR impact factor lacks the ability to discriminate different shapes of the citation distribution curves, whereas IF′ proposed in this paper does not have this drawback. In this regard, IF′ is an alternative to the JCR impact factor with unique features that reward journals with highly-cited papers, but disregard lowly-cited papers. We need to point out that whether journals with such peaked citation distribution are preferable to those having uniform distribution is arguable. However, current studies on the citation distributions show that the citation distributions obey the so-called power law^24^,^25^,^26^, stretched exponential^24^,^27^,^28^, lognormal^24^,^29^,^30^,^31^ and modified Bessel function^24^,^32^. According to the parameters involved in these functions, the citation distributions are usually skewed, rather than uniform.

In summary, IF′ is proposed as an alternative to the JCR impact factor with some unique features. (i) IF′ is designed to possess the ability to distinguish between different shapes of the citation distribution curves. It gives larger values of IF′ to those with relatively sharp citation distribution curves, whereas lower values to those with flat ones. This is in agreement with the common point of view of academic evaluation^33^,^34^. (ii) Citation information of three years is considered by IF′, rather than 2 years for JCR impact factor, and therefore IF′ carries more citation information. (iii) IF′ is basically consistent with the JCR impact factor, and the two impact factors have roughly the same order of magnitude. These features make IF′ relatively easy to be accepted by the academic community. IF′ may properly reflect the academic performance of a scientific journal in a way that is different from the JCR impact factor with some unique features that reward journals with highly cited papers, and therefore it could be used to complement the JCR impact factor.

## Methods

As an example, the journals in the category of biochemistry and molecular biology were studied. According to the Thomson Reuters Journal Citation Reports (JCR) Science Edition 2011, there are 290 journals listed in the subject category BIOCHEMISTRY & MOLECULAR BIOLOGY. Among these 290 journals, a few of them changed their titles during the period of 2006 to 2010. Some journals published fewer than 100 articles, whereas some were not continuously indexed by the Web of Science database (WoS) during the period of 2006–2010. All the journals mentioned above were excluded from the current study. Consequently, 227 journals were remained for the present study. We downloaded the Journal Summary List of the subject category BIOCHEMISTRY & MOLECULAR BIOLOGY in the JCR Science Edition 2011 and extracted the Impact Factor values of the 227 journals. The detailed titles and related data for the 227 journals are listed in the Appendix-1.

## Author Contributions

Conceived and designed the experiments: C.T.Z. Performed the experiments: Z.G.Y. Analyzed the data: C.T.Z. and Z.G.Y. Wrote the paper: C.T.Z.

## Supplementary Material

Supplementary Information Journal title, JCR impact factor and IF'

## Figures and Tables

**Table 1:** The front ten journals in the category of biochemistry and molecular biology in the alphabetic order^a^

No.	Full Journal Title	JCR IF	*C_total_*	*h*	*e*^2^	*t*^2^	*r*	IF′
1	ACS CHEMICAL BIOLOGY	6.446	1965	22	291	1190	0.495	10.879
2	ACTA BIOCHIMICA ET BIOPHYSICA SINICA	1.376	499	9	15	403	0.193	1.736
3	ACTA BIOCHIMICA POLONICA	1.491	359	8	42	253	0.407	3.260
4	ACTA CRYSTALLOGRAPHICA SECTION D	12.619	7491	16	6246	989	2.513	40.209
5	ACTA CRYSTALLOGRAPHICA SECTION F	0.506	905	7	23	833	0.166	1.163
6	ADDICTION BIOLOGY	4.833	717	15	138	354	0.624	9.365
7	AMERICAN JOURNAL OF RESPIRATORY CELL AND MOLECULAR BIOLOGY	5.125	2460	19	158	1941	0.285	5.421
8	AMINO ACIDS	3.248	2577	21	341	1795	0.436	9.153
9	AMYLOID-JOURNAL OF PROTEIN FOLDING DISORDERS	2.660	157	5	19	113	0.410	2.050
10	ANALYTICAL BIOCHEMISTRY	2.996	4230	20	156	3674	0.206	4.121

^a^*C_total_*, *h*, *e,* *t*, *r* and *IF*′ are the total citations, the *h*-index, *e*-index, *t*-index, the head-tail ratio and IF′, respectively, where *r* and *IF*′ are defined in eqs. (1) and (2).

**Table 2:** Comparison between two citation distributions for four pairs of journals with appropriately equal JCR *IF*, *h*-index and publication number^a^

	No.	Journal b	JCR IF	*h*	*C_h-core_*	*P*	*C_total_*	*e*^2^	*t*^2^	*r*	*IF*′
I	134	JLR	1.707	6	48	76	172	12	124	0.311	1.867
	147	JPB	1.711	6	78	83	191	42	113	0.610	3.658
II	74	CBP	1.923	10	116	316	920	16	804	0.141	1.411
	140	JMM	1.797	10	211	335	913	111	702	0.398	3.976
III	138	JME	2.274	11	167	214	801	46	634	0.269	2.963
	169	MB	2.171	11	222	212	702	101	480	0.459	5.046
IV	111	IJBM	2.453	14	246	360	1368	50	1122	0.211	2.955
	115	JB	2.371	14	371	375	1367	175	996	0.419	5.868

^a^The data are derived from the Appendix-1, where *C_h-core_*, *P*, *C_total,_* *e*, *t*, *r* and *IF*′ are the citations within the *h*-core, the number of publications, the total citations, the *e*-index, the *t*-index, the head-tail ratio, and IF′, respectively. The head-tail ratio *r* and *IF*′ are defined in eqs. (1) and (2), respectively.

^b^JLR, JPB, CBP, JMM, JME, MB, IJBM and JB denote the Journal of Liposome Research, Journal of Physiology and Biochemistry, Comparative Biochemistry and Physiology B-Biochemistry & Molecular Biology, Journal of Molecular Modeling, Journal of Molecular Evolution, Molecular Biotechnology, International Journal of Biological Macromolecules and Journal Of Biochemistry, respectively. The order numbers are in consistence with those in the Appendix-1.

**Table 3:** The top ten journals in the rankings based on IF′ and JCR impact factor^a^

No.	Full Journal Title	*IF*′	No.	Full Journal Title	JCR *IF*
1 (1)	CELL	57.411	1 (1)	CELL	32.403
2 (7)	ACTA CRYSTALLOGRAPHICA, SECTION D	40.209	2 (4)	NATURE MEDICINE	22.462
3 (15)	NUCLEIC ACIDS RESEARCH	36.984	3 (7)	NATURE CHEMICAL BIOLOGY	14.690
4 (2)	NATURE MEDICINE	32.167	4 (8)	MOLECULAR CELL	14.178
5 (6)	GENOME RESEARCH	24.595	5 (10)	MOLECULAR PSYCHIATRY	13.668
6 (12)	CELL DEATH AND DIFFERENTIATION	20.969	6 (5)	GENOME RESEARCH	13.608
7 (3)	NATURE CHEMICAL BIOLOGY	20.495	7 (2)	ACTA CRYSTALLOGRAPHICA, SECTION D	12.619
8 (4)	MOLECULAR CELL	17.437	8 (40)	CURRENT BIOLOGY	9.647
9 (9)	CURRENT OPINION IN STRUCTURAL BIOLOGY	16.234	9 (9)	CURRENT OPINION IN STRUCTURAL BIOLOGY	9.424
10 (5)	MOLECULAR PSYCHIATRY	15.392	10 (24)	EMBO JOURNAL	9.205

^a^The top ten journals in the rankings based on IF′ and the JCR impact factor are in the left and right side of the table. The integer within the parenthesis denotes the ranking order based on another index for the same journal. For example, on the left side of the table, the figures 3 (15) denote the fact that the journal Nucleic Acids Research is ranked No. 3 in the rankings based on IF′, whereas it is ranked No. 15 in the rankings based on the JCR impact factor.
