# Supplementary material for: A proposal for a novel impact factor as an alternative to the JCR impact factor
Source: Sci Rep. 2013 Dec 3;3:3410. doi: 10.1038/srep03410 (PMC3847704; doi:10.1038/srep03410)
Supplement: Supplementary Information — Journal title, JCR impact factor and IF' [file srep03410-s1.pdf]

Supplementary information

A proposal for a novel impact factor as an alternative to the JCR impact factor

Zu-Guo Yang[a] and Chun-Ting Zhang[b,*]

[a] Library, Tianjin University, Tianjin 300072, China
[b] Department of Physics, Tianjin University, Tianjin 300072, China

Appendix-1

No.	Full Journal Title	JCR IF	total citations	*h*-index	e^2	t^2	*e/t*	*IF'*
1	ACS CHEMICAL BIOLOGY	6.446	1965	22	291	1190	0.495	10.879
2	ACTA BIOCHIMICA ET BIOPHYSICA SINICA	1.376	499	9	15	403	0.193	1.736
3	ACTA BIOCHIMICA POLONICA	1.491	359	8	42	253	0.407	3.260
4	ACTA CRYSTALLOGRAPHICA SECTION D-BIOLOGICAL CRYSTALLOGRAPHY	12.619	7491	16	6246	989	2.513	40.209
5	ACTA CRYSTALLOGRAPHICA SECTION F-STRUCTURAL BIOLOGY AND CRYSTALLIZATION COMMUNICATIONS	0.506	905	7	23	833	0.166	1.163
6	ADDICTION BIOLOGY	4.833	717	15	138	354	0.624	9.365
7	AMERICAN JOURNAL OF RESPIRATORY CELL AND MOLECULAR BIOLOGY	5.125	2460	19	158	1941	0.285	5.421
8	AMINO ACIDS	3.248	2577	21	341	1795	0.436	9.153
9	AMYLOID-JOURNAL OF PROTEIN FOLDING DISORDERS	2.660	157	5	19	113	0.410	2.050
10	ANALYTICAL BIOCHEMISTRY	2.996	4230	20	156	3674	0.206	4.121
11	ANTIOXIDANTS & REDOX SIGNALING	8.456	4862	29	493	3528	0.374	10.841
12	APOPTOSIS	4.788	2073	20	301	1372	0.468	9.368
13	APPLIED BIOCHEMISTRY AND BIOTECHNOLOGY	1.943	2024	13	72	1783	0.201	2.612
14	ARCHIVES OF BIOCHEMISTRY AND BIOPHYSICS	2.935	2363	18	116	1923	0.246	4.421
15	ARCHIVES OF INSECT BIOCHEMISTRY AND PHYSIOLOGY	1.361	239	7	13	177	0.271	1.897
16	BIOCATALYSIS AND BIOTRANSFORMATION	0.905	147	5	22	100	0.469	2.345
17	BIOCHEMICAL AND BIOPHYSICAL RESEARCH COMMUNICATIONS	2.484	15879	28	334	14761	0.150	4.212
18	BIOCHEMICAL GENETICS	0.862	226	6	15	175	0.293	1.757
19	BIOCHEMICAL JOURNAL	4.897	7093	29	1159	5093	0.477	13.834
20	BIOCHEMICAL SOCIETY TRANSACTIONS	3.711	3475	22	346	2645	0.362	7.957
21	BIOCHEMICAL SYSTEMATICS AND ECOLOGY	0.931	415	6	14	365	0.196	1.175
22	BIOCHEMISTRY	3.422	13988	27	398	12861	0.176	4.750
23	BIOCHEMISTRY AND CELL BIOLOGY-BIOCHIMIE ET BIOLOGIE CELLULAIRE	2.673	828	14	138	494	0.529	7.400
24	BIOCHEMISTRY AND MOLECULAR BIOLOGY EDUCATION	0.840	106	3	8	89	0.300	0.899
25	BIOCHEMISTRY-MOSCOW	1.058	524	7	37	438	0.291	2.035
26	BIOCHIMICA ET BIOPHYSICA ACTA-BIOENERGETICS	4.843	3152	24	327	2249	0.381	9.151
27	BIOCHIMICA ET BIOPHYSICA ACTA-BIOMEMBRANES	3.990	3947	28	304	2859	0.326	9.130
28	BIOCHIMICA ET BIOPHYSICA ACTA-GENE REGULATORY MECHANISMS	4.405	1167	17	135	743	0.426	7.246
29	BIOCHIMICA ET BIOPHYSICA ACTA-GENERAL SUBJECTS	5.000	2854	25	429	1800	0.488	12.205
30	BIOCHIMICA ET BIOPHYSICA ACTA-MOLECULAR AND CELL BIOLOGY OF LIPIDS	5.269	2716	24	271	1869	0.381	9.139
31	BIOCHIMICA ET BIOPHYSICA ACTA-MOLECULAR BASIS OF DISEASE	5.387	2556	23	384	1643	0.483	11.119
32	BIOCHIMICA ET BIOPHYSICA ACTA-MOLECULAR CELL RESEARCH	5.538	3409	28	498	2127	0.484	13.548
33	BIOCHIMICA ET BIOPHYSICA ACTA-PROTEINS AND PROTEOMICS	3.635	2577	21	324	1812	0.423	8.880
34	BIOCHIMIE	3.022	1942	17	179	1474	0.348	5.924
35	BIOCONJUGATE CHEMISTRY	4.930	4344	24	284	3484	0.286	6.852
36	BIOELECTROCHEMISTRY	3.759	1143	16	85	802	0.326	5.209
37	BIOESSAYS	4.954	2031	21	219	1371	0.400	8.393
38	BIOFACTORS	4.933	920	16	138	526	0.512	8.195
39	BIOINORGANIC CHEMISTRY AND APPLICATIONS	0.716	57	3	7	41	0.413	1.240
40	BIOLOGICAL CHEMISTRY	2.965	1445	16	164	1025	0.400	6.400
41	BIOLOGICAL TRACE ELEMENT RESEARCH	1.923	929	10	32	797	0.200	2.004
42	BIOMACROMOLECULES	5.479	7541	28	469	6288	0.273	7.647
43	BIOMEDICAL CHROMATOGRAPHY	1.966	962	10	136	726	0.433	4.328
44	BIOMETALS	2.823	999	13	121	709	0.413	5.370
45	BIOORGANIC & MEDICINAL CHEMISTRY	2.921	8552	22	286	7782	0.192	4.218
46	BIOORGANIC CHEMISTRY	1.211	172	6	28	108	0.509	3.055
47	BIOPHYSICAL CHEMISTRY	2.203	988	11	36	831	0.208	2.290
48	BIOPOLYMERS	2.870	1467	14	117	1154	0.318	4.458
49	BIOSCIENCE BIOTECHNOLOGY AND BIOCHEMISTRY	1.276	2096	11	62	1913	0.180	1.980
50	BIOTECHNIQUES	2.669	712	14	131	385	0.583	8.166
51	BIOTECHNOLOGY AND APPLIED BIOCHEMISTRY	1.534	484	11	40	323	0.352	3.871
52	BMC MOLECULAR BIOLOGY	2.857	942	14	107	639	0.409	5.729
53	CANADIAN JOURNAL OF MICROBIOLOGY	1.363	617	9	18	518	0.186	1.678
54	CARBOHYDRATE RESEARCH	2.332	2701	16	89	2356	0.194	3.110
55	CELL	32.403	38205	94	7979	21390	0.611	57.411

56	CELL BIOCHEMISTRY AND BIOPHYSICS	3.743	452	12	151	157	0.981	11.768
57	CELL BIOCHEMISTRY AND FUNCTION	1.771	480	9	32	367	0.295	2.658
58	CELL DEATH AND DIFFERENTIATION	8.849	4910	33	1099	2722	0.635	20.969
59	CELLULAR & MOLECULAR BIOLOGY LETTERS	1.505	258	7	36	173	0.456	3.193
60	CELLULAR AND MOLECULAR BIOLOGY	0.975	357	12	25	188	0.365	4.376
61	CELLULAR AND MOLECULAR LIFE SCIENCES	6.570	6117	32	428	4665	0.303	9.693
62	CHANNELS	2.140	351	9	34	236	0.380	3.416
63	CHEMBIOCHEM	3.944	4188	23	175	3484	0.224	5.155
64	CHEMICAL BIOLOGY & DRUG DESIGN	2.282	1071	13	114	788	0.380	4.945
65	CHEMICAL SPECIATION AND BIOAVAILABILITY	0.397	40	2	3	33	0.302	0.603
66	CHEMICO-BIOLOGICAL INTERACTIONS	2.865	2887	16	139	2492	0.236	3.779
67	CHEMISTRY & BIODIVERSITY	1.804	1035	10	36	899	0.200	2.001
68	CHEMISTRY & BIOLOGY	5.829	2337	19	283	1693	0.409	7.768
69	CHEMISTRY AND PHYSICS OF LIPIDS	2.571	727	11	155	451	0.586	6.449
70	CHEMOECOLOGY	1.556	127	5	26	76	0.585	2.924
71	CHROMOSOMA	3.847	715	14	129	390	0.575	8.052
72	CHROMOSOME RESEARCH	3.087	806	14	116	494	0.485	6.784
73	COMPARATIVE BIOCHEMISTRY AND PHYSIOLOGY A-MOLECULAR & INTEGRATIVE PHYSIOLOGY	2.235	1547	14	69	1282	0.232	3.248
74	COMPARATIVE BIOCHEMISTRY AND PHYSIOLOGY B-BIOCHEMISTRY & MOLECULAR BIOLOGY	1.923	920	10	16	804	0.141	1.411
75	COMPARATIVE BIOCHEMISTRY AND PHYSIOLOGY C-TOXICOLOGY & PHARMACOLOGY	2.616	1191	13	62	960	0.254	3.304
76	COMPARATIVE BIOCHEMISTRY AND PHYSIOLOGY D-GENOMICS & PROTEOMICS	1.718	176	7	11	116	0.308	2.156
77	CURRENT BIOLOGY	9.647	11399	37	510	9520	0.231	8.564
78	CURRENT GENOMICS	2.408	420	9	48	291	0.406	3.655
79	CURRENT OPINION IN STRUCTURAL BIOLOGY	9.424	2790	26	593	1521	0.624	16.234
80	CYTOKINE	3.019	1484	16	89	1139	0.280	4.473
81	DIAGNOSTIC MOLECULAR PATHOLOGY	2.257	250	9	55	114	0.695	6.251
82	DNA AND CELL BIOLOGY	2.072	526	9	41	404	0.319	2.867
83	DOKLADY BIOCHEMISTRY AND BIOPHYSICS	0.326	85	3	9	67	0.367	1.100
84	EMBO JOURNAL	9.205	10529	40	506	8423	0.245	9.804
85	EMBO REPORTS	7.355	3154	25	381	2148	0.421	10.529
86	EUROPEAN CELLS & MATERIALS	3.028	277	8	45	168	0.518	4.140
87	EUROPEAN CYTOKINE NETWORK	1.726	169	6	18	115	0.396	2.374
88	EUROPEAN JOURNAL OF HUMAN GENETICS	4.400	3055	21	270	2344	0.339	7.127
89	EXPERIMENTAL AND MOLECULAR MEDICINE	2.481	753	11	72	560	0.359	3.944
90	EXTREMOPHILES	2.941	629	12	63	422	0.386	4.637
91	FEBS JOURNAL	3.790	6411	25	243	5543	0.209	5.234
92	FEBS LETTERS	3.538	7916	27	354	6833	0.228	6.146
93	FISH PHYSIOLOGY AND BIOCHEMISTRY	1.528	400	7	32	319	0.317	2.217
94	FLY	1.296	212	8	28	120	0.483	3.864
95	FOLIA HISTOCHEMICA ET CYTOBIOLOGICA	0.807	217	6	15	166	0.301	1.804
96	FREE RADICAL BIOLOGY AND MEDICINE	5.423	6460	29	628	4991	0.355	10.287
97	FREE RADICAL RESEARCH	2.878	1066	12	98	824	0.345	4.138
98	GENE THERAPY	3.710	1920	18	103	1493	0.263	4.728
99	GENERAL PHYSIOLOGY AND BIOPHYSICS	1.192	260	6	50	174	0.536	3.216
100	GENETICS AND MOLECULAR BIOLOGY	0.634	221	5	10	186	0.232	1.159
101	GENETICS AND MOLECULAR RESEARCH	1.184	633	8	29	540	0.232	1.854
102	GENOME RESEARCH	13.608	9533	46	1649	5768	0.535	24.595
103	GLYCOBIOLOGY	3.580	1743	17	105	1349	0.279	4.743
104	GLYCOCONJUGATE JOURNAL	2.117	629	10	74	455	0.403	4.033
105	HEMOGLOBIN	1.304	220	6	28	156	0.424	2.542
106	HUMAN MOLECULAR GENETICS	7.636	11704	36	740	9668	0.277	9.960
107	INDIAN JOURNAL OF BIOCHEMISTRY & BIOPHYSICS	1.142	210	7	21	140	0.387	2.711
108	INSECT BIOCHEMISTRY AND MOLECULAR BIOLOGY	3.246	1057	13	59	829	0.267	3.468
109	INSECT MOLECULAR BIOLOGY	2.529	774	12	43	587	0.271	3.248
110	INTERNATIONAL JOURNAL OF BIOCHEMISTRY & CELL BIOLOGY	4.634	4644	27	476	3439	0.372	10.045
111	INTERNATIONAL JOURNAL OF BIOLOGICAL MACROMOLECULES	2.453	1368	14	50	1122	0.211	2.955
112	INTERNATIONAL JOURNAL OF BIOLOGICAL SCIENCES	2.699	622	11	136	365	0.610	6.715
113	INTERNATIONAL JOURNAL OF PEPTIDE RESEARCH AND THERAPEUTICS	0.986	127	5	7	95	0.271	1.357
114	IUBMB LIFE	3.514	1271	17	122	860	0.377	6.403

115	JOURNAL OF BIOCHEMICAL AND MOLECULAR TOXICOLOGY	1.380	188	7	17	122	0.373	2.613
116	JOURNAL OF BIOCHEMISTRY	2.371	1367	14	175	996	0.419	5.868
117	JOURNAL OF BIOLOGICAL CHEMISTRY	4.773	62098	45	1218	58855	0.144	6.474
118	JOURNAL OF BIOLOGICAL INORGANIC CHEMISTRY	3.289	1226	15	138	863	0.400	5.998
119	JOURNAL OF BIOMOLECULAR NMR	3.612	1099	14	250	653	0.619	8.662
120	JOURNAL OF CARBOHYDRATE CHEMISTRY	0.631	83	4	10	57	0.419	1.675
121	JOURNAL OF CELLULAR BIOCHEMISTRY	2.868	3965	20	275	3290	0.289	5.782
122	JOURNAL OF CHEMICAL ECOLOGY	2.657	1214	14	83	935	0.298	4.171
123	JOURNAL OF CHEMICAL NEUROANATOMY	2.435	441	9	55	305	0.425	3.822
124	JOURNAL OF COMPUTER-AIDED MOLECULAR DESIGN	3.386	786	13	84	533	0.397	5.161
125	JOURNAL OF ENZYME INHIBITION AND MEDICINAL CHEMISTRY	1.617	730	9	55	594	0.304	2.739
126	JOURNAL OF EVOLUTIONARY BIOCHEMISTRY AND PHYSIOLOGY	0.243	13	2	1	8	0.354	0.707
127	JOURNAL OF FOOD BIOCHEMISTRY	0.815	177	5	4	148	0.164	0.822
128	JOURNAL OF GENETICS AND GENOMICS	1.883	432	10	58	274	0.460	4.601
129	JOURNAL OF INORGANIC BIOCHEMISTRY	3.354	2120	16	121	1743	0.263	4.216
130	JOURNAL OF INTEGRATIVE PLANT BIOLOGY	2.534	791	12	88	559	0.397	4.761
131	JOURNAL OF INTERFERON AND CYTOKINE RESEARCH	3.063	771	14	179	396	0.672	9.413
132	JOURNAL OF LIPID RESEARCH	5.559	6470	32	551	4895	0.336	10.736
133	JOURNAL OF LIPOSOME RESEARCH	1.707	172	6	12	124	0.311	1.867
134	JOURNAL OF MEMBRANE BIOLOGY	1.808	478	9	15	382	0.198	1.783
135	JOURNAL OF MOLECULAR BIOLOGY	4.001	11985	28	255	10946	0.153	4.274
136	JOURNAL OF MOLECULAR CATALYSIS B-ENZYMATIC	2.735	2102	16	124	1722	0.268	4.294
137	JOURNAL OF MOLECULAR EVOLUTION	2.274	801	11	46	634	0.269	2.963
138	JOURNAL OF MOLECULAR GRAPHICS & MODELLING	2.184	641	10	66	475	0.373	3.728
139	JOURNAL OF MOLECULAR MODELING	1.797	913	10	111	702	0.398	3.976
140	JOURNAL OF MOLECULAR NEUROSCIENCE	2.504	892	12	27	721	0.194	2.322
141	JOURNAL OF MOLECULAR RECOGNITION	3.310	523	9	96	346	0.527	4.741
142	JOURNAL OF NEUROCHEMISTRY	4.061	8254	28	340	7130	0.218	6.114
143	JOURNAL OF NUTRITIONAL BIOCHEMISTRY	3.891	1538	16	119	1163	0.320	5.118
144	JOURNAL OF PEPTIDE SCIENCE	1.799	616	10	34	482	0.266	2.656
145	JOURNAL OF PHOTOCHEMISTRY AND PHOTOBIOLOGY B-BIOLOGY	2.814	1050	14	114	740	0.392	5.495
146	JOURNAL OF PHYSIOLOGY AND BIOCHEMISTRY	1.711	191	6	42	113	0.610	3.658
147	JOURNAL OF PLANT BIOCHEMISTRY AND BIOTECHNOLOGY	0.523	73	4	12	45	0.516	2.066
148	JOURNAL OF RECEPTORS AND SIGNAL TRANSDUCTION	1.588	201	6	14	151	0.304	1.827
149	JOURNAL OF STEROID BIOCHEMISTRY AND MOLECULAR BIOLOGY	3.053	1748	15	85	1438	0.243	3.647
150	JOURNAL OF STRUCTURAL BIOLOGY	3.406	1870	16	86	1528	0.237	3.796
151	JOURNAL OF THE AMERICAN SOCIETY FOR MASS SPECTROMETRY	4.002	3384	21	208	2735	0.276	5.791
152	JOURNAL OF TRACE ELEMENTS IN MEDICINE AND BIOLOGY	1.683	220	7	12	159	0.275	1.923
153	JOURNAL OF ZHEJIANG UNIVERSITY-SCIENCE B	1.099	415	6	28	351	0.282	1.695
154	LIPIDS	2.129	754	11	61	572	0.327	3.592
155	LIPIDS IN HEALTH AND DISEASE	2.170	545	10	36	409	0.297	2.967
156	LUMINESCENCE	1.731	256	7	8	199	0.201	1.404
157	MACROMOLECULAR BIOSCIENCE	3.886	1720	15	172	1323	0.361	5.408
158	MAGNESIUM RESEARCH	1.519	113	6	5	72	0.264	1.581
159	MAMMALIAN GENOME	2.887	601	12	54	403	0.366	4.393
160	MATRIX BIOLOGY	3.299	621	11	49	451	0.330	3.626
161	METHODS	4.011	1718	19	148	1209	0.350	6.648
162	MOLECULAR AND BIOCHEMICAL PARASITOLOGY	2.551	1131	13	103	859	0.346	4.502
163	MOLECULAR AND CELLULAR BIOLOGY	5.527	9487	31	424	8102	0.229	7.092
164	MOLECULAR AND CELLULAR PROBES	2.078	380	9	36	263	0.370	3.330
165	MOLECULAR BIOLOGY	0.658	140	4	1	123	0.090	0.361
166	MOLECULAR BIOLOGY AND EVOLUTION	5.550	4479	25	443	3411	0.360	9.010
167	MOLECULAR BIOLOGY REPORTS	2.929	2898	14	40	2662	0.123	1.716
168	MOLECULAR BIOSYSTEMS	3.534	2620	19	185	2074	0.299	5.675
169	MOLECULAR BIOTECHNOLOGY	2.171	702	11	101	480	0.459	5.046
170	MOLECULAR CANCER	3.993	2294	16	69	1969	0.187	2.995
171	MOLECULAR CARCINOGENESIS	3.164	1184	13	133	882	0.388	5.048
172	MOLECULAR CELL	14.178	14092	50	1257	10335	0.349	17.437
173	MOLECULAR ECOLOGY	5.522	7253	28	397	6072	0.256	7.160
174	MOLECULAR GENETICS AND GENOMICS	2.635	792	12	67	581	0.340	4.075
175	MOLECULAR GENETICS AND METABOLISM	3.193	1637	14	83	1358	0.247	3.461
176	MOLECULAR IMMUNOLOGY	2.897	3407	17	137	2981	0.214	3.644
177	MOLECULAR MEDICINE	3.757	660	12	97	419	0.481	5.774
178	MOLECULAR MEMBRANE BIOLOGY	2.863	326	10	53	173	0.553	5.535

179	MOLECULAR MICROBIOLOGY	5.010	7147	24	209	6362	0.181	4.350
180	MOLECULAR PHYLOGENETICS AND EVOLUTION	3.609	4147	20	126	3621	0.187	3.731
181	MOLECULAR PLANT	5.546	1786	17	120	1377	0.295	5.018
182	MOLECULAR PLANT-MICROBE INTERACTIONS	4.431	2115	18	190	1601	0.344	6.201
183	MOLECULAR PSYCHIATRY	13.668	4047	32	568	2455	0.481	15.392
184	MOLECULAR REPRODUCTION AND DEVELOPMENT	2.532	964	12	62	758	0.286	3.432
185	MOLECULAR SYSTEMS BIOLOGY	8.626	2517	24	245	1696	0.380	9.122
186	MOLECULAR VISION	2.205	2061	14	35	1830	0.138	1.936
187	MOLECULES AND CELLS	2.178	1080	13	77	834	0.304	3.950
188	NATURE CHEMICAL BIOLOGY	14.690	6188	40	954	3634	0.512	20.495
189	NATURE MEDICINE	22.462	12013	59	1955	6577	0.545	32.167
190	NEUROCHEMICAL RESEARCH	2.240	1847	15	162	1460	0.333	4.997
191	NEUROCHEMISTRY INTERNATIONAL	2.857	2095	15	169	1701	0.315	4.728
192	NITRIC OXIDE-BIOLOGY AND CHEMISTRY	3.548	792	13	138	485	0.533	6.934
193	NUCLEIC ACIDS RESEARCH	8.026	28443	62	6456	18143	0.597	36.984
194	NUCLEOSIDES NUCLEOTIDES & NUCLEIC ACIDS	0.899	241	7	9	183	0.222	1.552
195	OLIGONUCLEOTIDES	2.797	321	9	107	133	0.897	8.073
196	ONCOGENE	6.373	9815	34	638	8021	0.282	9.589
197	PEPTIDES	2.434	2691	17	139	2263	0.248	4.213
198	PESTICIDE BIOCHEMISTRY AND PHYSIOLOGY	1.713	472	8	26	382	0.261	2.087
199	PHOTOCHEMICAL & PHOTOBIOLOGICAL SCIENCES	2.584	1603	13	64	1370	0.216	2.810
200	PHOTOCHEMISTRY AND PHOTOBIOLOGY	2.413	1422	12	213	1065	0.447	5.367
201	PHYTOCHEMISTRY LETTERS	1.222	193	6	11	146	0.274	1.647
202	PLANT CELL	8.987	7597	34	379	6062	0.250	8.501
203	PLANT MOLECULAR BIOLOGY	4.150	2065	19	283	1421	0.446	8.479
204	PLANT SCIENCE	2.945	1485	15	131	1129	0.341	5.110
205	PREPARATIVE BIOCHEMISTRY & BIOTECHNOLOGY	0.466	64	3	4	51	0.280	0.840
206	PROCESS BIOCHEMISTRY	2.627	1989	14	92	1701	0.233	3.256
207	PROGRESS IN BIOCHEMISTRY AND BIOPHYSICS	0.554	244	4	2	226	0.094	0.376
208	PROSTAGLANDINS & OTHER LIPID MEDIATORS	2.705	459	11	84	254	0.575	6.326
209	PROSTAGLANDINS LEUKOTRIENES AND ESSENTIAL FATTY ACIDS	3.367	910	14	146	568	0.507	7.098
210	PROTEIN AND PEPTIDE LETTERS	1.942	1217	14	239	782	0.553	7.740
211	PROTEIN ENGINEERING DESIGN & SELECTION	2.937	958	13	98	691	0.377	4.896
212	PROTEIN EXPRESSION AND PURIFICATION	1.587	1064	11	36	907	0.199	2.191
213	PROTEIN JOURNAL	1.038	211	5	34	152	0.473	2.365
214	PROTEIN SCIENCE	2.798	2223	16	113	1854	0.247	3.950
215	PROTEINS-STRUCTURE FUNCTION AND BIOINFORMATICS	3.392	3899	23	289	3081	0.306	7.044
216	PROTEOMICS	4.505	6424	28	304	5336	0.239	6.683
217	REDOX REPORT	1.732	181	6	13	132	0.314	1.883
218	RNA BIOLOGY	4.933	946	15	60	661	0.301	4.519
219	RNA-A PUBLICATION OF THE RNA SOCIETY	5.095	4034	26	367	2991	0.350	9.107
220	RUSSIAN JOURNAL OF BIOORGANIC CHEMISTRY	0.636	146	5	9	112	0.283	1.417
221	SCIENCE SIGNALING	7.499	4007	30	457	2650	0.415	12.458
222	STEROIDS	2.829	1358	15	121	1012	0.346	5.187
223	STRUCTURE	6.347	3263	24	331	2356	0.375	8.996
224	TRACE ELEMENTS AND ELECTROLYTES	0.469	35	3	3	23	0.361	1.083
225	TRANSGENIC RESEARCH	2.754	661	11	38	502	0.275	3.026
226	TURKISH JOURNAL OF BIOCHEMISTRY-TURK BIYOKIMYA DERGISI	0.258	29	3	1	19	0.229	0.688
227	YEAST	1.895	342	8	19	259	0.271	2.167

Appendix-2

IF' Rank	JCR-*IF* Rank	Full Journal Title	*IF'*	JCR-*IF* Rank	*IF'* Rank	Full Journal Title	JCR *IF*
1	1	CELL	57.411	1	1	CELL	32.403
2	7	ACTA CRYSTALLOGRAPHICA SECTION D-BIOLOGICAL CRYSTALLOGRAPHY	40.209	2	4	NATURE MEDICINE	22.462
3	15	NUCLEIC ACIDS RESEARCH	36.984	3	7	NATURE CHEMICAL BIOLOGY	14.690
4	2	NATURE MEDICINE	32.167	4	8	MOLECULAR CELL	14.178
5	6	GENOME RESEARCH	24.595	5	10	MOLECULAR PSYCHIATRY	13.668
6	12	CELL DEATH AND DIFFERENTIATION	20.969	6	5	GENOME RESEARCH	13.608
7	3	NATURE CHEMICAL BIOLOGY	20.495	7	2	ACTA CRYSTALLOGRAPHICA SECTION D-BIOLOGICAL CRYSTALLOGRAPHY	12.619
8	4	MOLECULAR CELL	17.437	8	40	CURRENT BIOLOGY	9.647
9	9	CURRENT OPINION IN STRUCTURAL BIOLOGY	16.234	9	9	CURRENT OPINION IN STRUCTURAL BIOLOGY	9.424
10	5	MOLECULAR PSYCHIATRY	15.392	10	24	EMBO JOURNAL	9.205
11	42	BIOCHEMICAL JOURNAL	13.834	11	41	PLANT CELL	8.987
12	27	BIOCHIMICA ET BIOPHYSICA ACTA-MOLECULAR CELL RESEARCH	13.548	12	6	CELL DEATH AND DIFFERENTIATION	8.849
13	17	SCIENCE SIGNALING	12.458	13	34	MOLECULAR SYSTEMS BIOLOGY	8.626
14	37	BIOCHIMICA ET BIOPHYSICA ACTA-GENERAL SUBJECTS	12.205	14	18	ANTIOXIDANTS & REDOX SIGNALING	8.456
15	66	CELL BIOCHEMISTRY AND BIOPHYSICS	11.768	15	3	NUCLEIC ACIDS RESEARCH	8.026
16	32	BIOCHIMICA ET BIOPHYSICA ACTA-MOLECULAR BASIS OF DISEASE	11.119	16	23	HUMAN MOLECULAR GENETICS	7.636
17	20	ACS CHEMICAL BIOLOGY	10.879	17	13	SCIENCE SIGNALING	7.499
18	14	ANTIOXIDANTS & REDOX SIGNALING	10.841	18	20	EMBO REPORTS	7.355
19	24	JOURNAL OF LIPID RESEARCH	10.736	19	25	CELLULAR AND MOLECULAR LIFE SCIENCES	6.570
20	18	EMBO REPORTS	10.529	20	17	ACS CHEMICAL BIOLOGY	6.446
21	31	FREE RADICAL BIOLOGY AND MEDICINE	10.287	21	26	ONCOGENE	6.373
22	47	INTERNATIONAL JOURNAL OF BIOCHEMISTRY & CELL BIOLOGY	10.045	22	37	STRUCTURE	6.347
23	16	HUMAN MOLECULAR GENETICS	9.960	23	49	CHEMISTRY & BIOLOGY	5.829
24	10	EMBO JOURNAL	9.804	24	19	JOURNAL OF LIPID RESEARCH	5.559
25	19	CELLULAR AND MOLECULAR LIFE SCIENCES	9.693	25	36	MOLECULAR BIOLOGY AND EVOLUTION	5.550
26	21	ONCOGENE	9.589	26	97	MOLECULAR PLANT	5.546
27	91	JOURNAL OF INTERFERON AND CYTOKINE RESEARCH	9.413	27	12	BIOCHIMICA ET BIOPHYSICA ACTA-MOLECULAR CELL RESEARCH	5.538
28	45	APOPTOSIS	9.368	28	57	MOLECULAR AND CELLULAR BIOLOGY	5.527
29	44	ADDICTION BIOLOGY	9.365	29	54	MOLECULAR ECOLOGY	5.522
30	86	AMINO ACIDS	9.153	30	51	BIOMACROMOLECULES	5.479
31	43	BIOCHIMICA ET BIOPHYSICA ACTA-BIOENERGETICS	9.151	31	21	FREE RADICAL BIOLOGY AND MEDICINE	5.423
32	33	BIOCHIMICA ET BIOPHYSICA ACTA-MOLECULAR AND CELL BIOLOGY OF LIPIDS	9.139	32	16	BIOCHIMICA ET BIOPHYSICA ACTA-MOLECULAR BASIS OF DISEASE	5.387
33	58	BIOCHIMICA ET BIOPHYSICA ACTA-BIOMEMBRANES	9.130	33	32	BIOCHIMICA ET BIOPHYSICA ACTA-MOLECULAR AND CELL BIOLOGY OF LIPIDS	5.269
34	13	MOLECULAR SYSTEMS BIOLOGY	9.122	34	84	AMERICAN JOURNAL OF RESPIRATORY CELL AND MOLECULAR BIOLOGY	5.125
35	35	RNA-A PUBLICATION OF THE RNA SOCIETY	9.107	35	35	RNA-A PUBLICATION OF THE RNA SOCIETY	5.095
36	25	MOLECULAR BIOLOGY AND EVOLUTION	9.010	36	116	MOLECULAR MICROBIOLOGY	5.010
37	22	STRUCTURE	8.996	37	14	BIOCHIMICA ET BIOPHYSICA ACTA-GENERAL SUBJECTS	5.000
38	69	BIOCHIMICA ET BIOPHYSICA ACTA-PROTEINS AND PROTEOMICS	8.880	38	43	BIOESSAYS	4.954
39	70	JOURNAL OF BIOMOLECULAR NMR	8.662	39	44	BIOFACTORS	4.933
40	8	CURRENT BIOLOGY	8.564	40	109	RNA BIOLOGY	4.933
41	11	PLANT CELL	8.501	41	60	BIOCONJUGATE CHEMISTRY	4.930
42	52	PLANT MOLECULAR BIOLOGY	8.479	42	11	BIOCHEMICAL JOURNAL	4.897
43	38	BIOESSAYS	8.393	43	31	BIOCHIMICA ET BIOPHYSICA ACTA-BIOENERGETICS	4.843
44	39	BIOFACTORS	8.195	44	29	ADDICTION BIOLOGY	4.833

45	123	BIOTECHNIQUES	8.166	45	28	APOPTOSIS	4.788
46	117	OLIGONUCLEOTIDES	8.073	46	65	JOURNAL OF BIOLOGICAL CHEMISTRY	4.773
47	62	CHROMOSOMA	8.052	47	22	INTERNATIONAL JOURNAL OF BIOCHEMISTRY & CELL BIOLOGY	4.634
48	67	BIOCHEMICAL SOCIETY TRANSACTIONS	7.957	48	63	PROTEOMICS	4.505
49	23	CHEMISTRY & BIOLOGY	7.768	49	71	MOLECULAR PLANT-MICROBE INTERACTIONS	4.431
50	163	PROTEIN AND PEPTIDE LETTERS	7.740	50	53	BIOCHIMICA ET BIOPHYSICA ACTA-GENE REGULATORY MECHANISMS	4.405
51	30	BIOMACROMOLECULES	7.647	51	55	EUROPEAN JOURNAL OF HUMAN GENETICS	4.400
52	122	BIOCHEMISTRY AND CELL BIOLOGY-BIOCHIMIE ET BIOLOGIE CELLULAIRE	7.400	52	42	PLANT MOLECULAR BIOLOGY	4.150
53	50	BIOCHIMICA ET BIOPHYSICA ACTA-GENE REGULATORY MECHANISMS	7.246	53	73	JOURNAL OF NEUROCHEMISTRY	4.061
54	29	MOLECULAR ECOLOGY	7.160	54	64	METHODS	4.011
55	51	EUROPEAN JOURNAL OF HUMAN GENETICS	7.127	55	77	JOURNAL OF THE AMERICAN SOCIETY FOR MASS SPECTROMETRY	4.002
56	81	PROSTAGLANDINS LEUKOTRIENES AND ESSENTIAL FATTY ACIDS	7.098	56	119	JOURNAL OF MOLECULAR BIOLOGY	4.001
57	28	MOLECULAR AND CELLULAR BIOLOGY	7.092	57	162	MOLECULAR CANCER	3.993
58	79	PROTEINS-STRUCTURE FUNCTION AND BIOINFORMATICS	7.044	58	33	BIOCHIMICA ET BIOPHYSICA ACTA-BIOMEMBRANES	3.990
59	73	NITRIC OXIDE-BIOLOGY AND CHEMISTRY	6.934	59	92	CHEMBIOCHEM	3.944
60	41	BIOCONJUGATE CHEMISTRY	6.852	60	93	JOURNAL OF NUTRITIONAL BIOCHEMISTRY	3.891
61	90	CHROMOSOME RESEARCH	6.784	61	85	MACROMOLECULAR BIOSCIENCE	3.886
62	121	INTERNATIONAL JOURNAL OF BIOLOGICAL SCIENCES	6.715	62	47	CHROMOSOMA	3.847
63	48	PROTEOMICS	6.683	63	88	FEBS JOURNAL	3.790
64	54	METHODS	6.648	64	89	BIOELECTROCHEMISTRY	3.759
65	46	JOURNAL OF BIOLOGICAL CHEMISTRY	6.474	65	79	MOLECULAR MEDICINE	3.757
66	130	CHEMISTRY AND PHYSICS OF LIPIDS	6.449	66	15	CELL BIOCHEMISTRY AND BIOPHYSICS	3.743
67	76	IUBMB LIFE	6.403	67	48	BIOCHEMICAL SOCIETY TRANSACTIONS	3.711
68	97	BIOLOGICAL CHEMISTRY	6.400	68	106	GENE THERAPY	3.710
69	120	PROSTAGLANDINS & OTHER LIPID MEDIATORS	6.326	69	38	BIOCHIMICA ET BIOPHYSICA ACTA-PROTEINS AND PROTEOMICS	3.635
70	147	DIAGNOSTIC MOLECULAR PATHOLOGY	6.251	70	39	JOURNAL OF BIOMOLECULAR NMR	3.612
71	49	MOLECULAR PLANT-MICROBE INTERACTIONS	6.201	71	139	MOLECULAR PHYLOGENETICS AND EVOLUTION	3.609
72	74	FEBS LETTERS	6.146	72	103	GLYCOBIOLOGY	3.580
73	53	JOURNAL OF NEUROCHEMISTRY	6.114	73	59	NITRIC OXIDE-BIOLOGY AND CHEMISTRY	3.548
74	85	JOURNAL OF BIOLOGICAL INORGANIC CHEMISTRY	5.998	74	72	FEBS LETTERS	3.538
75	94	BIOCHIMIE	5.924	75	81	MOLECULAR BIOSYSTEMS	3.534
76	143	JOURNAL OF BIOCHEMISTRY	5.868	76	67	IUBMB LIFE	3.514
77	55	JOURNAL OF THE AMERICAN SOCIETY FOR MASS SPECTROMETRY	5.791	77	102	BIOCHEMISTRY	3.422
78	108	JOURNAL OF CELLULAR BIOCHEMISTRY	5.782	78	137	JOURNAL OF STRUCTURAL BIOLOGY	3.406
79	65	MOLECULAR MEDICINE	5.774	79	58	PROTEINS-STRUCTURE FUNCTION AND BIOINFORMATICS	3.392
80	111	BMC MOLECULAR BIOLOGY	5.729	80	91	JOURNAL OF COMPUTER-AIDED MOLECULAR DESIGN	3.386
81	75	MOLECULAR BIOSYSTEMS	5.675	81	56	PROSTAGLANDINS LEUKOTRIENES AND ESSENTIAL FATTY ACIDS	3.367
82	110	MOLECULAR MEMBRANE BIOLOGY	5.535	82	121	JOURNAL OF INORGANIC BIOCHEMISTRY	3.354
83	115	JOURNAL OF PHOTOCHEMISTRY AND PHOTOBIOLOGY B-BIOLOGY	5.495	83	104	JOURNAL OF MOLECULAR RECOGNITION	3.310
84	34	AMERICAN JOURNAL OF RESPIRATORY CELL AND MOLECULAR BIOLOGY	5.421	84	145	MATRIX BIOLOGY	3.299
85	61	MACROMOLECULAR BIOSCIENCE	5.408	85	74	JOURNAL OF BIOLOGICAL INORGANIC CHEMISTRY	3.289
86	114	BIOMETALS	5.370	86	30	AMINO ACIDS	3.248
87	141	PHOTOCHEMISTRY AND PHOTOBIOLOGY	5.367	87	147	INSECT BIOCHEMISTRY AND MOLECULAR BIOLOGY	3.246
88	63	FEBS JOURNAL	5.234	88	148	MOLECULAR GENETICS AND METABOLISM	3.193

89	64	BIOELECTROCHEMISTRY	5.209	89	95	MOLECULAR CARCINOGENESIS	3.164
90	113	STEROIDS	5.187	90	61	CHROMOSOME RESEARCH	3.087
91	80	JOURNAL OF COMPUTER-AIDED MOLECULAR DESIGN	5.161	91	27	JOURNAL OF INTERFERON AND CYTOKINE RESEARCH	3.063
92	59	CHEMBIOCHEM	5.155	92	143	JOURNAL OF STEROID BIOCHEMISTRY AND MOLECULAR BIOLOGY	3.053
93	60	JOURNAL OF NUTRITIONAL BIOCHEMISTRY	5.118	93	125	EUROPEAN CELLS & MATERIALS	3.028
94	98	PLANT SCIENCE	5.110	94	75	BIOCHIMIE	3.022
95	89	MOLECULAR CARCINOGENESIS	5.048	95	111	CYTOKINE	3.019
96	154	MOLECULAR BIOTECHNOLOGY	5.046	96	127	ANALYTICAL BIOCHEMISTRY	2.996
97	26	MOLECULAR PLANT	5.018	97	68	BIOLOGICAL CHEMISTRY	2.965
98	148	NEUROCHEMICAL RESEARCH	4.997	98	94	PLANT SCIENCE	2.945
99	145	CHEMICAL BIOLOGY & DRUG DESIGN	4.945	99	107	EXTREMOPHILES	2.941
100	100	PROTEIN ENGINEERING DESIGN & SELECTION	4.896	100	100	PROTEIN ENGINEERING DESIGN & SELECTION	2.937
101	132	JOURNAL OF INTEGRATIVE PLANT BIOLOGY	4.761	101	113	ARCHIVES OF BIOCHEMISTRY AND BIOPHYSICS	2.935
102	77	BIOCHEMISTRY	4.750	102	203	MOLECULAR BIOLOGY REPORTS	2.929
103	72	GLYCOBIOLOGY	4.743	103	120	BIOORGANIC & MEDICINAL CHEMISTRY	2.921
104	83	JOURNAL OF MOLECULAR RECOGNITION	4.741	104	144	MOLECULAR IMMUNOLOGY	2.897
105	112	NEUROCHEMISTRY INTERNATIONAL	4.728	105	114	MAMMALIAN GENOME	2.887
106	68	GENE THERAPY	4.728	106	126	FREE RADICAL RESEARCH	2.878
107	99	EXTREMOPHILES	4.637	107	112	BIOPOLYMERS	2.870
108	167	JOURNAL OF GENETICS AND GENOMICS	4.601	108	78	JOURNAL OF CELLULAR BIOCHEMISTRY	2.868
109	40	RNA BIOLOGY	4.519	109	138	CHEMICO-BIOLOGICAL INTERACTIONS	2.865
110	131	MOLECULAR AND BIOCHEMICAL PARASITOLOGY	4.502	110	82	MOLECULAR MEMBRANE BIOLOGY	2.863
111	95	CYTOKINE	4.473	111	80	BMC MOLECULAR BIOLOGY	2.857
112	107	BIOPOLYMERS	4.458	112	105	NEUROCHEMISTRY INTERNATIONAL	2.857
113	101	ARCHIVES OF BIOCHEMISTRY AND BIOPHYSICS	4.421	113	90	STEROIDS	2.829
114	105	MAMMALIAN GENOME	4.393	114	86	BIOMETALS	2.823
115	206	CELLULAR AND MOLECULAR BIOLOGY	4.376	115	83	JOURNAL OF PHOTOCHEMISTRY AND PHOTOBIOLOGY B-BIOLOGY	2.814
116	36	MOLECULAR MICROBIOLOGY	4.350	116	132	PROTEIN SCIENCE	2.798
117	161	BIOMEDICAL CHROMATOGRAPHY	4.328	117	46	OLIGONUCLEOTIDES	2.797
118	119	JOURNAL OF MOLECULAR CATALYSIS B-ENZYMATIC	4.294	118	161	TRANSGENIC RESEARCH	2.754
119	56	JOURNAL OF MOLECULAR BIOLOGY	4.274	119	118	JOURNAL OF MOLECULAR CATALYSIS B-ENZYMATIC	2.735
120	103	BIOORGANIC & MEDICINAL CHEMISTRY	4.218	120	69	PROSTAGLANDINS & OTHER LIPID MEDIATORS	2.705
121	82	JOURNAL OF INORGANIC BIOCHEMISTRY	4.216	121	62	INTERNATIONAL JOURNAL OF BIOLOGICAL SCIENCES	2.699
122	140	PEPTIDES	4.213	122	52	BIOCHEMISTRY AND CELL BIOLOGY-BIOCHIMIE ET BIOLOGIE CELLULAIRE	2.673
123	136	BIOCHEMICAL AND BIOPHYSICAL RESEARCH COMMUNICATIONS	4.212	123	45	BIOTECHNIQUES	2.669
124	125	JOURNAL OF CHEMICAL ECOLOGY	4.171	124	187	AMYLOID-JOURNAL OF PROTEIN FOLDING DISORDERS	2.660
125	93	EUROPEAN CELLS & MATERIALS	4.140	125	124	JOURNAL OF CHEMICAL ECOLOGY	2.657
126	106	FREE RADICAL RESEARCH	4.138	126	128	MOLECULAR GENETICS AND GENOMICS	2.635
127	96	ANALYTICAL BIOCHEMISTRY	4.121	127	154	PROCESS BIOCHEMISTRY	2.627
128	126	MOLECULAR GENETICS AND GENOMICS	4.075	128	152	COMPARATIVE BIOCHEMISTRY AND PHYSIOLOGY C-TOXICOLOGY & PHARMACOLOGY	2.616
129	158	GLYCOCONJUGATE JOURNAL	4.033	129	168	PHOTOCHEMICAL & PHOTOBIOLOGICAL SCIENCES	2.584
130	171	JOURNAL OF MOLECULAR MODELING	3.976	130	66	CHEMISTRY AND PHYSICS OF LIPIDS	2.571
131	153	MOLECULES AND CELLS	3.950	131	110	MOLECULAR AND BIOCHEMICAL PARASITOLOGY	2.551
132	116	PROTEIN SCIENCE	3.950	132	101	JOURNAL OF INTEGRATIVE PLANT BIOLOGY	2.534
133	137	EXPERIMENTAL AND MOLECULAR MEDICINE	3.944	133	149	MOLECULAR REPRODUCTION AND DEVELOPMENT	2.532

134	185	BIOTECHNOLOGY AND APPLIED BIOCHEMISTRY	3.871	134	156	INSECT MOLECULAR BIOLOGY	2.529
135	195	FLY	3.864	135	179	JOURNAL OF MOLECULAR NEUROSCIENCE	2.504
136	139	JOURNAL OF CHEMICAL NEUROANATOMY	3.822	136	123	BIOCHEMICAL AND BIOPHYSICAL RESEARCH COMMUNICATIONS	2.484
137	78	JOURNAL OF STRUCTURAL BIOLOGY	3.796	137	133	EXPERIMENTAL AND MOLECULAR MEDICINE	2.481
138	109	CHEMICO-BIOLOGICAL INTERACTIONS	3.779	138	165	INTERNATIONAL JOURNAL OF BIOLOGICAL MACROMOLECULES	2.453
139	71	MOLECULAR PHYLOGENETICS AND EVOLUTION	3.731	139	136	JOURNAL OF CHEMICAL NEUROANATOMY	2.435
140	152	JOURNAL OF MOLECULAR GRAPHICS & MODELLING	3.728	140	122	PEPTIDES	2.434
141	178	JOURNAL OF PHYSIOLOGY AND BIOCHEMISTRY	3.658	141	87	PHOTOCHEMISTRY AND PHOTOBIOLOGY	2.413
142	142	CURRENT GENOMICS	3.655	142	142	CURRENT GENOMICS	2.408
143	92	JOURNAL OF STEROID BIOCHEMISTRY AND MOLECULAR BIOLOGY	3.647	143	76	JOURNAL OF BIOCHEMISTRY	2.371
144	104	MOLECULAR IMMUNOLOGY	3.644	144	159	CARBOHYDRATE RESEARCH	2.332
145	84	MATRIX BIOLOGY	3.626	145	99	CHEMICAL BIOLOGY & DRUG DESIGN	2.282
146	157	LIPIDS	3.592	146	164	JOURNAL OF MOLECULAR EVOLUTION	2.274
147	87	INSECT BIOCHEMISTRY AND MOLECULAR BIOLOGY	3.468	147	70	DIAGNOSTIC MOLECULAR PATHOLOGY	2.257
148	88	MOLECULAR GENETICS AND METABOLISM	3.461	148	98	NEUROCHEMICAL RESEARCH	2.240
149	133	MOLECULAR REPRODUCTION AND DEVELOPMENT	3.432	149	155	COMPARATIVE BIOCHEMISTRY AND PHYSIOLOGY A-MOLECULAR & INTEGRATIVE PHYSIOLOGY	2.235
150	156	CHANNELS	3.416	150	192	MOLECULAR VISION	2.205
151	159	MOLECULAR AND CELLULAR PROBES	3.330	151	180	BIOPHYSICAL CHEMISTRY	2.203
152	128	COMPARATIVE BIOCHEMISTRY AND PHYSIOLOGY C-TOXICOLOGY & PHARMACOLOGY	3.304	152	140	JOURNAL OF MOLECULAR GRAPHICS & MODELLING	2.184
153	189	ACTA BIOCHIMICA POLONICA	3.260	153	131	MOLECULES AND CELLS	2.178
154	127	PROCESS BIOCHEMISTRY	3.256	154	96	MOLECULAR BIOTECHNOLOGY	2.171
155	149	COMPARATIVE BIOCHEMISTRY AND PHYSIOLOGY A-MOLECULAR & INTEGRATIVE PHYSIOLOGY	3.248	155	163	LIPIDS IN HEALTH AND DISEASE	2.170
156	134	INSECT MOLECULAR BIOLOGY	3.248	156	150	CHANNELS	2.140
157	199	GENERAL PHYSIOLOGY AND BIOPHYSICS	3.216	157	146	LIPIDS	2.129
158	188	CELLULAR & MOLECULAR BIOLOGY LETTERS	3.193	158	129	GLYCOCONJUGATE JOURNAL	2.117
159	144	CARBOHYDRATE RESEARCH	3.110	159	151	MOLECULAR AND CELLULAR PROBES	2.078
160	198	BIOORGANIC CHEMISTRY	3.055	160	167	DNA AND CELL BIOLOGY	2.072
161	118	TRANSGENIC RESEARCH	3.026	161	117	BIOMEDICAL CHROMATOGRAPHY	1.966
162	57	MOLECULAR CANCER	2.995	162	174	APPLIED BIOCHEMISTRY AND BIOTECHNOLOGY	1.943
163	155	LIPIDS IN HEALTH AND DISEASE	2.967	163	50	PROTEIN AND PEPTIDE LETTERS	1.942
164	146	JOURNAL OF MOLECULAR EVOLUTION	2.963	164	189	BIOLOGICAL TRACE ELEMENT RESEARCH	1.923
165	138	INTERNATIONAL JOURNAL OF BIOLOGICAL MACROMOLECULES	2.955	165	211	COMPARATIVE BIOCHEMISTRY AND PHYSIOLOGY B-BIOCHEMISTRY & MOLECULAR BIOLOGY	1.923
166	184	CHEMOECOLOGY	2.924	166	183	YEAST	1.895
167	160	DNA AND CELL BIOLOGY	2.867	167	108	JOURNAL OF GENETICS AND GENOMICS	1.883
168	129	PHOTOCHEMICAL & PHOTOBIOLOGICAL SCIENCES	2.810	168	200	JOURNAL OF MEMBRANE BIOLOGY	1.808
169	181	JOURNAL OF ENZYME INHIBITION AND MEDICINAL CHEMISTRY	2.739	169	190	CHEMISTRY & BIODIVERSITY	1.804
170	201	INDIAN JOURNAL OF BIOCHEMISTRY & BIOPHYSICS	2.711	170	172	JOURNAL OF PEPTIDE SCIENCE	1.799
171	172	CELL BIOCHEMISTRY AND FUNCTION	2.658	171	130	JOURNAL OF MOLECULAR MODELING	1.797
172	170	JOURNAL OF PEPTIDE SCIENCE	2.656	172	171	CELL BIOCHEMISTRY AND FUNCTION	1.771
173	190	JOURNAL OF BIOCHEMICAL AND MOLECULAR TOXICOLOGY	2.613	173	195	REDOX REPORT	1.732
174	162	APPLIED BIOCHEMISTRY AND BIOTECHNOLOGY	2.612	174	212	LUMINESCENCE	1.731
175	194	HEMOGLOBIN	2.542	175	176	EUROPEAN CYTOKINE NETWORK	1.726

176	175	EUROPEAN CYTOKINE NETWORK	2.374	176	184	COMPARATIVE BIOCHEMISTRY AND PHYSIOLOGY D-GENOMICS & PROTEOMICS	1.718
177	204	PROTEIN JOURNAL	2.365	177	185	PESTICIDE BIOCHEMISTRY AND PHYSIOLOGY	1.713
178	208	BIOCATALYSIS AND BIOTRANSFORMATION	2.345	178	141	JOURNAL OF PHYSIOLOGY AND BIOCHEMISTRY	1.711
179	135	JOURNAL OF MOLECULAR NEUROSCIENCE	2.322	179	196	JOURNAL OF LIPOSOME RESEARCH	1.707
180	151	BIOPHYSICAL CHEMISTRY	2.290	180	193	JOURNAL OF TRACE ELEMENTS IN MEDICINE AND BIOLOGY	1.683
181	186	FISH PHYSIOLOGY AND BIOCHEMISTRY	2.217	181	169	JOURNAL OF ENZYME INHIBITION AND MEDICINAL CHEMISTRY	1.617
182	183	PROTEIN EXPRESSION AND PURIFICATION	2.191	182	198	JOURNAL OF RECEPTORS AND SIGNAL TRANSDUCTION	1.588
183	166	YEAST	2.167	183	182	PROTEIN EXPRESSION AND PURIFICATION	1.587
184	176	COMPARATIVE BIOCHEMISTRY AND PHYSIOLOGY D-GENOMICS & PROTEOMICS	2.156	184	166	CHEMOECOLOGY	1.556
185	177	PESTICIDE BIOCHEMISTRY AND PHYSIOLOGY	2.087	185	134	BIOTECHNOLOGY AND APPLIED BIOCHEMISTRY	1.534
186	220	JOURNAL OF PLANT BIOCHEMISTRY AND BIOTECHNOLOGY	2.066	186	181	FISH PHYSIOLOGY AND BIOCHEMISTRY	1.528
187	124	AMYLOID-JOURNAL OF PROTEIN FOLDING DISORDERS	2.050	187	208	MAGNESIUM RESEARCH	1.519
188	203	BIOCHEMISTRY-MOSCOW	2.035	188	158	CELLULAR & MOLECULAR BIOLOGY LETTERS	1.505
189	164	BIOLOGICAL TRACE ELEMENT RESEARCH	2.004	189	153	ACTA BIOCHIMICA POLONICA	1.491
190	169	CHEMISTRY & BIODIVERSITY	2.001	190	173	JOURNAL OF BIOCHEMICAL AND MOLECULAR TOXICOLOGY	1.380
191	196	BIOSCIENCE BIOTECHNOLOGY AND BIOCHEMISTRY	1.980	191	202	ACTA BIOCHIMICA ET BIOPHYSICA SINICA	1.376
192	150	MOLECULAR VISION	1.936	192	205	CANADIAN JOURNAL OF MICROBIOLOGY	1.363
193	180	JOURNAL OF TRACE ELEMENTS IN MEDICINE AND BIOLOGY	1.923	193	194	ARCHIVES OF INSECT BIOCHEMISTRY AND PHYSIOLOGY	1.361
194	193	ARCHIVES OF INSECT BIOCHEMISTRY AND PHYSIOLOGY	1.897	194	175	HEMOGLOBIN	1.304
195	173	REDOX REPORT	1.883	195	135	FLY	1.296
196	179	JOURNAL OF LIPOSOME RESEARCH	1.867	196	191	BIOSCIENCE BIOTECHNOLOGY AND BIOCHEMISTRY	1.276
197	200	GENETICS AND MOLECULAR RESEARCH	1.854	197	207	PHYTOCHEMISTRY LETTERS	1.222
198	182	JOURNAL OF RECEPTORS AND SIGNAL TRANSDUCTION	1.827	198	160	BIOORGANIC CHEMISTRY	1.211
199	213	FOLIA HISTOCHEMICA ET CYTOBIOLOGICA	1.804	199	157	GENERAL PHYSIOLOGY AND BIOPHYSICS	1.192
200	168	JOURNAL OF MEMBRANE BIOLOGY	1.783	200	197	GENETICS AND MOLECULAR RESEARCH	1.184
201	210	BIOCHEMICAL GENETICS	1.757	201	170	INDIAN JOURNAL OF BIOCHEMISTRY & BIOPHYSICS	1.142
202	191	ACTA BIOCHIMICA ET BIOPHYSICA SINICA	1.736	202	204	JOURNAL OF ZHEJIANG UNIVERSITY-SCIENCE B	1.099
203	102	MOLECULAR BIOLOGY REPORTS	1.716	203	188	BIOCHEMISTRY-MOSCOW	1.058
204	202	JOURNAL OF ZHEJIANG UNIVERSITY-SCIENCE B	1.695	204	177	PROTEIN JOURNAL	1.038
205	192	CANADIAN JOURNAL OF MICROBIOLOGY	1.678	205	213	INTERNATIONAL JOURNAL OF PEPTIDE RESEARCH AND THERAPEUTICS	0.986
206	218	JOURNAL OF CARBOHYDRATE CHEMISTRY	1.675	206	115	CELLULAR AND MOLECULAR BIOLOGY	0.975
207	197	PHYTOCHEMISTRY LETTERS	1.647	207	215	BIOCHEMICAL SYSTEMATICS AND ECOLOGY	0.931
208	187	MAGNESIUM RESEARCH	1.581	208	178	BIOCATALYSIS AND BIOTRANSFORMATION	0.905
209	209	NUCLEOSIDES NUCLEOTIDES & NUCLEIC ACIDS	1.552	209	209	NUCLEOSIDES NUCLEOTIDES & NUCLEIC ACIDS	0.899
210	216	RUSSIAN JOURNAL OF BIOORGANIC CHEMISTRY	1.417	210	201	BIOCHEMICAL GENETICS	0.862
211	165	COMPARATIVE BIOCHEMISTRY AND PHYSIOLOGY B-BIOCHEMISTRY & MOLECULAR BIOLOGY	1.411	211	220	BIOCHEMISTRY AND MOLECULAR BIOLOGY EDUCATION	0.840
212	174	LUMINESCENCE	1.404	212	222	JOURNAL OF FOOD BIOCHEMISTRY	0.815
213	205	INTERNATIONAL JOURNAL OF PEPTIDE RESEARCH AND THERAPEUTICS	1.357	213	199	FOLIA HISTOCHEMICA ET CYTOBIOLOGICA	0.807
214	214	BIOINORGANIC CHEMISTRY AND	1.240	214	214	BIOINORGANIC CHEMISTRY AND	0.716

		APPLICATIONS				APPLICATIONS	
215	207	BIOCHEMICAL SYSTEMATICS AND ECOLOGY	1.175	215	227	MOLECULAR BIOLOGY	0.658
216	221	ACTA CRYSTALLOGRAPHICA SECTION F-STRUCTURAL BIOLOGY AND CRYSTALLIZATION COMMUNICATIONS	1.163	216	210	RUSSIAN JOURNAL OF BIOORGANIC CHEMISTRY	0.636
217	217	GENETICS AND MOLECULAR BIOLOGY	1.159	217	217	GENETICS AND MOLECULAR BIOLOGY	0.634
218	225	DOKLADY BIOCHEMISTRY AND BIOPHYSICS	1.100	218	206	JOURNAL OF CARBOHYDRATE CHEMISTRY	0.631
219	222	TRACE ELEMENTS AND ELECTROLYTES	1.083	219	226	PROGRESS IN BIOCHEMISTRY AND BIOPHYSICS	0.554
220	211	BIOCHEMISTRY AND MOLECULAR BIOLOGY EDUCATION	0.899	220	186	JOURNAL OF PLANT BIOCHEMISTRY AND BIOTECHNOLOGY	0.523
221	223	PREPARATIVE BIOCHEMISTRY & BIOTECHNOLOGY	0.840	221	216	ACTA CRYSTALLOGRAPHICA SECTION F-STRUCTURAL BIOLOGY AND CRYSTALLIZATION COMMUNICATIONS	0.506
222	212	JOURNAL OF FOOD BIOCHEMISTRY	0.822	222	219	TRACE ELEMENTS AND ELECTROLYTES	0.469
223	227	JOURNAL OF EVOLUTIONARY BIOCHEMISTRY AND PHYSIOLOGY	0.707	223	221	PREPARATIVE BIOCHEMISTRY & BIOTECHNOLOGY	0.466
224	226	TURKISH JOURNAL OF BIOCHEMISTRY-TURK BIYOKIMYA DERGISI	0.688	224	225	CHEMICAL SPECIATION AND BIOAVAILABILITY	0.397
225	224	CHEMICAL SPECIATION AND BIOAVAILABILITY	0.603	225	218	DOKLADY BIOCHEMISTRY AND BIOPHYSICS	0.326
226	219	PROGRESS IN BIOCHEMISTRY AND BIOPHYSICS	0.376	226	224	TURKISH JOURNAL OF BIOCHEMISTRY-TURK BIYOKIMYA DERGISI	0.258
227	215	MOLECULAR BIOLOGY	0.361	227	223	JOURNAL OF EVOLUTIONARY BIOCHEMISTRY AND PHYSIOLOGY	0.243
